# Interviewing for residency positions while completing a graduate degree: Considerations for graduate students, mentors, and program directors

**DOI:** 10.1002/acm2.13700

**Published:** 2022-06-14

**Authors:** Wayne Newhauser, Garrett Pitcher, Christine Swanson, Samuel G. Armato, Hania Al‐Hallaq

**Affiliations:** ^1^ Louisiana State University, Nicholson Hall Baton Rouge Louisiana USA; ^2^ Mary Bird Perkins Cancer Center Baton Rouge Louisiana USA; ^3^ University of Louisville, James G Brown Cancer Center Louisville Kentucky USA; ^4^ University of Chicago Chicago Illinois USA

**Keywords:** bottleneck, interviewing, medical physics graduate education, residency training, resiliency

The pipeline of new clinical medical physicists in the United States includes graduate education followed by residency training. A major bottleneck occurs as students on the verge of completing their advanced degree programs seek positions in residency training programs; for example, in 2022 between a quarter and a half of applicants did not enter residency training.[1] The origin of the bottleneck was the introduction in 2014 of the American Board of Radiology's eligibility requirement for professional board certification that mandated the completion of an accredited residency program.^1^ The bottleneck acutely impacts graduate students. Impacts of concern include anxiety and depression, which are prevalent in graduate students across a spectrum of disciplines in the United States.^2^ Other potential impacts include shortages of qualified medical physicists, which could in turn impact the quality, safety, and availability of patient care.^3^, ^4^, ^5^, ^6^, ^7^

Although there is literature on navigating the medical physics education and training pathways,^8^, ^9^, ^10^ there is little guidance for graduate students in balancing their limited time to be spent on the competing tasks of interviewing for a residency position and simultaneously completing their degree. In principle, this should be quite simple: Each student interviews with enough residency programs to successfully secure an offer of admission to at least one program, but the number of interviews should not impact the completion of the graduate degree. In practice, the bottleneck problem makes it difficult for a student to estimate this number with confidence. Many students have managed this uncertainty by applying to, and interviewing with, large numbers of residency programs.^11^ Excessive time spent interviewing, however, can negatively impact students, faculty, and education and training programs. A recent study^12^ suggests that the bottleneck problem could contribute to stress and burnout. For all these reasons, we seek to raise an awareness of key factors that students and their faculty mentors should consider as they navigate through the bottleneck. Solutions to the bottleneck are beyond the scope of this editorial; rather, we seek to provide information and guidance to facilitate navigating the system, as it currently exists. Guidance is provided as suggestions for consideration, and they should not be construed as requirements.

To make well‐informed decisions, it is helpful to consider outcomes, ranging from ideal to suboptimal. The hallmarks of an ideal pipeline would be exclusively good outcomes:

- All students complete their degrees on time.
- All students who are admitted to a residency training program begin on schedule.
- Students and programs spend an appropriate amount of time interviewing; this time should be sufficient to make informed and wise decisions but not excessive to distract from other responsibilities.
- All residency programs utilize their full training capacity by successfully recruiting residents to all open residency positions.
- All qualified students who wish to enter a residency training program are able to do so, and all residency graduates subsequently enter the clinical medical physics workforce.[2]

The specific outcomes to reduce in severity and/or frequency include the following:

- Late completion of a degree.
- Deferral or cancellation of admission to residency program due to delay in degree completion.
- Failure of qualified students to gain admission to a residency training program.
- Vacant training positions in accredited residency programs (10 of 133, or 7%, of positions to begin in 2022 were unmatched).^13^
- Anxiety and depression of students due to the residency interview process.

We believe that students, faculty advisors, and program directors should make decisions that are well informed, rational, and responsible. Here we provide information we believe will be helpful to all stakeholders.

- In 2020, 189 candidates were admitted to residency programs (147 to therapy programs and 42 to imaging programs). Overall, 44% and 35% held MS and PhD degrees, respectively, from accredited medical physics programs, and 21% held certificates or other qualifications.^11^ In 2022, the number of applicants participating in the National Match Service (NMS) match exceeded the number of positions offered by a large margin,^13^ as discussed earlier.
- Most students apply to a large number of programs (>10) and interview with a subset. In 2022, the NMS reported that students seeking residency ranked an average of 10 programs^13^ (students rank programs after concluding their interviews). Evidently, the large numbers of interviews are driven, at least in part, by the students’ hope that participating in more interviews will increase their chance of matching.
- The Medical Physics Residency Application Program (MP‐RAP), which is a separate service provided by the American Association of Physicists in Medicine, requires an applicant to pay for a copy of their application to be sent to residency programs.^14^ The service is sold in increments of 10 application submissions.
- The time commitment required to submit the written application depends on whether students choose to apply to programs within the national match system, to programs outside of it, or both.
- The student time spent “per interview” varies from about 30 min for a screening interview to 3.5 h for a full interview. This excludes time spent for preparations, travel, receptions, tours, and any post‐interview follow‐up items.
- Many advisors did not complete residency training programs and therefore lack the personal experience or current knowledge needed to best advise their students on applying to residency programs.
- Residency programs have variable start dates, commonly 1 July, that can work to the student's advantage or disadvantage.

*Guidance to graduate students*:

- Communicate with your faculty mentor(s) regarding your intention to apply for residency at least 1 year prior. Communicate regularly and openly throughout the process. Seek feedback from your mentor on your application materials well in advance of submission deadlines.
- Take ownership, make a plan and schedule, and apply time‐management skills throughout the process to ensure you meet all commitments and deadlines.
- Prioritize your academic and professional commitments (e.g., graduation, research, and teaching, service).
- In developing a plan, consider the time spent per application and interview and the total time available for these activities.
- Budget the time necessary for interview preparation, travel, screening interviews, full interviews, and follow‐up conversations.
- Be prepared to explain and justify your plans to your advisor (some may have less knowledge of the residency application process than a student might assume).
- Complete degree requirements (including research) on time.
- Practice your interviewing skills prior to residency interviews. Thoughtfully set an appropriate number of applications and interviews. Twenty applications have been the average for most students.^15^ As these are quick to prepare (compared to interviewing), the number of applications is relevant but not critical in terms of time management. In contrast, interviewing is more time‐consuming, and considerable planning and subjective judgment may be required to ascertain an appropriate number of interviews.
- Practice your scientific presentation skills prior to residency interviews. Many programs require interviewees to deliver an oral presentation, for example, on thesis research projects.
- In budgeting the total time for interviews, take into consideration your competitiveness. This will depend on your qualifications, the past performance of your graduate program (i.e., placement statistics), and the demand for residency programs (i.e., admission statistics) to which you apply. Composited statistics are available on program websites and elsewhere.^16^
- Create contingency plans in case you are not offered admission to a residency training program. This could include acquiring additional clinical experience (e.g., medical physicist assistant) or research training (e.g., postdoctoral fellowship). Such activities may improve competitiveness in future applications to residency programs. Other examples include seeking employment in nonclinical careers in medical physics (e.g., in industry and government).^17^
- Prioritize applications and interviews with programs that offer training opportunities that best prepare you to succeed in reaching your particular career goals (e.g., an exclusively clinical focus vs. combination of clinical and academic emphases).
- Participate in residency fairs to learn about residency programs from current residents, graduates, and faculty.
- Maintain your physical and mental health and your resiliency through proper diet, exercise, and sleep habits. Self‐care is essential.

*Guidance to faculty advisors of graduate students*:

- Explain that on‐time degree completion is essential.
- Faculty advisors and and/or graduate programs should consider providing interview training and/or advice to their students.
- Be candid with students about your level of knowledge of the residency application process.
- Assist students in understanding program placement rates and how that may impact the number and types of programs to which they should apply.
- Be reasonable in accommodating a student's justified need to spend time interviewing. This may vary by programs, institutions, and students. We estimate that 2–4 weeks of cumulative time would be sufficient for most students.
- Set expectations upfront regarding leave, namely, the graduate student's use of vacation time and/or the faculty advisor authorizing time to interview during the regular hours of a student's graduate assistantship.
- Set deadlines for the completion of each major degree requirement, such as research and manuscript submissions. This should be done proactively and in collaboration with the student in order to ensure on‐time degree completion and to accommodate time spent interviewing and related tasks. Consider formalizing an individual development plan process for students early in their course of study, with annual reviews.

*Guidance to residency program directors*:

- The time spent per applicant and the total time spent on all applicants should be considered, including time spent interviewing.
- Post your program's most recently available composited statistics (e.g., application and admission rates), in accordance with accreditation requirements, so prospective applicants may assess their competitiveness and prospects for admission to your program.
- Provide prospective applicants with an estimate of the time commitments expected to complete various steps in the program's application process through admission (e.g., screening interview, full‐length interview, visitation, tour, and follow‐up conversation). This helps students to appropriately balance time spent on completing their degrees and interviewing.
- Consider using screening interviews (e.g., by videoconference) to conserve time, cost, and other impacts of long‐distance travel. When possible, the scheduling of screening interviews should accommodate students’ preexisting commitments (e.g., teaching or research assistantship duties).
- Share your program's interview schedule through the Society of Directors of Academic Medical Physics Programs’ calendar (https://www.sdampp.org/calendar.php).^18^ This calendar is open access and facilitates efficient scheduling and avoidance of scheduling conflicts between residency programs.

Making good choices regarding one's education, training, and career is complex under the best of circumstances. Currently, a large number of applicants are not able to enter residency training programs. Navigating these turbulent waters calls for well‐informed, rational, and responsible decision‐making by students, mentors, graduate education programs, and residency training programs. Personalized decision‐making is essential because the characteristics and goals of individual students and programs are diverse. We discourage prescriptive approaches, such as recommending a maximum number of interviews per student or per residency opening. Lastly, we recommend continued annual surveillance by the education community to monitor this serious and evolving situation.

## CONFLICT OF INTEREST

The authors declare that there is no conflict of interest that could be perceived as prejudicing the impartiality of the material reported.

## AUTHOR CONTRIBUTION

Wayne Newhauser drafted the manuscript and provided project leadership. Each of the coauthors contributed intellectual content and edited the manuscript.

## Footnotes

1 For the intents and purposes of this editorial, we require only a crude bracket around the true value, which is not known. We took data on the MedPhys Match (run by National Matching Services [NMS] for positions beginning in 2022).^13^ These data reveal that 133 of 264 (50%) of applicants matched to a residency. However, only 214 applicants participated in the match, and of those only 180 were ranked by at least one program (NMS calls this subset ranked applicants “acceptable” applicants; we use this term for consistency; be noted th‐at we are unable to confirm that ranked applicants are acceptable or unranked applicants are unacceptable). A total of 133 of 180 (74%) of acceptable applicants matched to a residency program. Hence, these data suggest a bracket spanning 50%–74% of applicants, depending on the denominator selected. For comparison, according to the Commission on Accreditation of Medical Physics Education Programs (CAMPEP), 323 students graduated from accredited programs in 2020 (a figure which excludes nine graduates from DMP programs),^19^ of which at least 50% applied to residency programs. We speculate that the reason for which the AAPM's value is larger than that from CAMPEP is that the former includes a backlog of frustrated applicants (who completed their degree in previous years and are attempting admission a second or higher time), whereas the latter excludes these individuals.

2 To reach this nirvanic state, graduate degree program admissions in a given year would have to equal future demand for residency graduates (in each subspecialty), for example, 4–5 years hence for MS degree holders, 7 or more years for PhD holders, and 1–2 years for postdoctoral certificate holders. Graduate admissions targets and workforce projections are challenging problems that are beyond the remit of this editorial.

## References

[1] The American Board of Radiology, Initial Certification for Medical Physics . Accessed May 12, 2022. https://www.theabr.org/medical‐physics/initial‐certification/part‐2‐exam/requirements‐application

[2] Satinsky EN , Kimura T , Kiang MV , et al. Systematic review and meta‐analysis of depression, anxiety, and suicidal ideation among Ph.D. students. Sci Rep. 2021;11(1):14370. 10.1038/s41598-021-93687-7 34257319 PMC8277873

[3] Newhauser WD . The medical physics workforce. Health Phys. 2017;112(2):139‐148. 28027152 10.1097/HP.0000000000000614

[4] Swanson C . An Evaluation of the Supply and Demand of Radiation Oncology Medical Physicists in the United States. University of Louisville; 2019. 10.18297/etd/3224

[5] Jordan DW , Newhauser WD , Mills MD . Current state of the imaging physics workforce and financial model. J Appl Clin Med Phys. 2021;22(12):4‐6. 10.1002/acm2.13489 PMC8664146 34890479

[6] Newhauser WD , Williams JP , Noska MA , et al. The professional radiation workforce. J Appl Clin Med Phys. (in review). 10.1002/acm2.13848 PMC9880970 36705250

[7] Newhauser WD , Gress DA , Mills MD , et al. The medical physics workforce. J Appl Clin Med Phys. (in review). 10.1002/acm2.13762 PMC9880968 36705248

[8] Silverstein E , Burmeister J , Fullerton G . SDAMPP Student Guide to a Medical Physics Career. Society of Directors of Academic Medical Physics Programs; 2016. https://www.sdampp.org/documents/SDAMPPStudentGuideToAMedicalPhysicsCareer.pdf

[9] Loughery B , Starkschall G , Hendrickson K , et al. Navigating the medical physics education and training landscape. J Appl Clin Med Phys. 2017;18(6):275‐287. 10.1002/acm2.12202 29125231 PMC5689917

[10] Antolak JA . Matchmaker, matchmaker, find me a match. J Appl Clin Med Phys. 2015;16(1):5425. 10.1120/jacmp.v16i1.5425 25679181 PMC5689979

[11] Dugan N . CAMPEP Residency Program Report. Accessed May 11, 2022. https://www.campep.org/2020AnnualResidencyReport.pdf

[12] Paradis KC , Ryan KA , Schmid S , et al. A qualitative investigation of resilience and well‐being among medical physics residents. J Appl Clin Med Phys. 2022;23(3):e13554. 10.1002/acm2.13554 35128786 PMC8906227

[13] National Matching Services . Summary Results of the Medphys Match for Positions Beginning in 2022. Accessed May 11, 2022. https://natmatch.com/medphys/stats/2022stats.pdf

[14] Medical Physics Residency Application Program. American Association of Physicists in Medicine, Accessed May 11, 2022. https://mprap.aapm.org/

[15] Hendrickson KRG , Juang T , Rodrigues AE , Burmeister JW . The MedPhys match survey: search criteria and advice for programs and applicants. J Appl Clin Med Phys. 2021;22(5):150‐167. 10.1002/acm2.13235 PMC8130228 33786983

[16] Highlights of the 2020 Medphys match, Education Council Report. *AAPM Newsletter* . July/August 2020: 45, 4. Accessed May 11, 2022. https://issuu.com/aapmdocs/docs/4504?mode=embed&viewMode=doublePage&backgroundColor=eeeeee

[17] SDAMPP . Advising Students on Non‐Clinical Careers. Society of Directors of Academic Medical Physics Programs. Accessed May 11, 2022. https://www.sdampp.org//2021OctoberWebinar.php.2021

[18] SDAMPP . Online interview calendar. Society of Directors of Academic Medical Physicis Programs. Accessed May 11, 2022. https://www.sdampp.org/calendar.php

[19] Hintenlang D . CAMPEP Graduate Program Report. Comission on the Accreditation of Medical Physics Education Programs. Accessed May 11, 2022. http://www.campep.org/2020AnnualGraduateReport.pdf

